# Aggressive Trimodality Therapy for T1N2M1 Nonsmall Cell Lung Cancer with Synchronous Solitary Brain Metastasis: Case Report and Rationale

**DOI:** 10.1155/2009/276571

**Published:** 2010-02-09

**Authors:** Timothy N. Showalter, Alexander Lin

**Affiliations:** ^1^Department of Radiation Oncology, Jefferson Medical College, Kimmel Cancer Center, Thomas Jefferson University, 111 S. 11th Street, Philadelphia, PA 19107, USA; ^2^Department of Radiation Oncology, University of Pennsylvania, 3400 Spruce Street, 2 Donner Building, Philadelphia, PA 19104, USA

## Abstract

Aggressive treatment, including resection of both metastasis and primary tumor, has been studied for non-small cell lung cancer patients with synchronous solitary brain metastasis. Involvement of mediastinal lymph nodes is considered a poor prognostic factor and a contraindication to surgical resection of the primary lung tumor after treatment for brain metastasis. Here we present the case of a patient who presented with a Stage IV T1N2M1 non-small cell lung cancer with synchronous solitary brain metastasis. He is alive and without evidence of disease two years after aggressive, multimodality treatment that included craniotomy, whole-brain radiation therapy, thoracic surgery, chemotherapy, and mediastinal radiation therapy.

## 1. Introduction

Brain metastases affect approximately 25% of patients with non-small cell lung cancer (NSCLC). For the subset of patients who present with a solitary brain metastasis (SBM), in the absence of other sites of metastatic disease, aggressive local therapy to the primary and metastasis is warranted [1]. Craniotomy for tumor resection and stereotactic radiosurgery (SRS) are widely accepted components of therapy for patients with SBM from NSCLC [1–4]. Several retrospective studies support the surgical resection of the primary tumor for NSCLC patients who present with synchronous, solitary brain metastasis [5–8]. In most series, patients with negative lymph nodes have better outcomes than patients with node-positive disease [6, 8–10]. In fact, the American College of Chest Physicians (ACCP) guidelines consider mediastinal lymph node involvement to be a contraindication to resection of the primary tumor for NSCLC with SBM [1]. However, the current case report is presented as an example of long-term survival after aggressive, multimodality therapy for an NSCLC patient with synchronous SBM and N2 nodal disease.

## 2. Case Presentation

**Figure 1:**
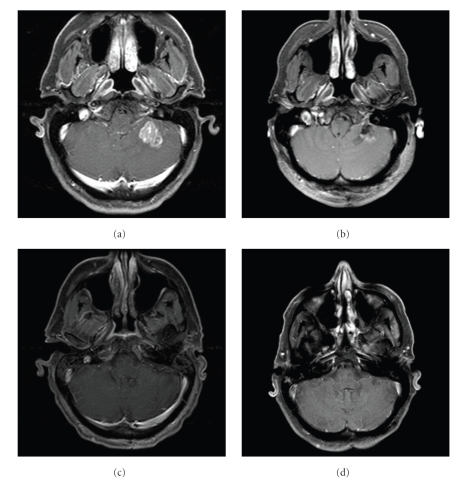
Axial, T1-weighted, postcontrast MR images of the brain. (a) A 2.3 × 2.3 × 2.5 cm left cerebellar lesion occurred synchronous with the diagnosis of a primary NSCLC. (b) The left cerebellar lesion was completely removed through a left suboccipital craniectomy. (c) No residual enhancement is noted after WBRT. (d) There is no evidence of recurrent brain metastasis on MRI obtained 2 years after diagnosis.

A 52-year-old male presented to medical attention with severe headaches. Magnetic resonance imaging (MRI) revealed an enhancing, intra-axial mass in the left cerebellum, measuring 2.3 × 2.3 × 2.5 cm and accompanied by surrounding edema (Figure 1(a)). A CT of the chest, abdomen, and pelvis was performed, revealing a left upper lobe lung mass with ipsilateral hilar and mediastinal lymphadenopathy. The suspected clinical diagnosis was lung cancer with brain metastasis. He underwent a suboccipital craniectomy for removal of the brain lesion. Postoperative MRI demonstrated gross total resection of the brain metastasis (Figure 1(b)). Pathology showed metastatic, poorly differentiated adenocarcinoma, with immunohistochemical findings consistent with a primary lung tumor (TTF-1 and CK7 positive). Whole-brain radiation therapy (WBRT) was administered postoperatively, consisting of 2.5 Gy daily fractions to 37.5 Gy, delivered with opposed lateral portals.

He was evaluated in multidisciplinary lung clinic after WBRT for discussion of further management of his NSCLC. His past medical history was significant for only hypercholesterolemia and history of a benign right flank mass that was surgically removed in the remote past. He had a thirty-pack-year history of smoking and chose to quit smoking when diagnosed with NSCLC. On examination, his lungs were clear to auscultation bilaterally. No focal neurologic deficits were discovered on examination. His Karnofsky Performance Status (KPS) was 100%, with an absence of any adverse prognostic factors such as weight loss or laboratory abnormalities.

**Figure 2:**
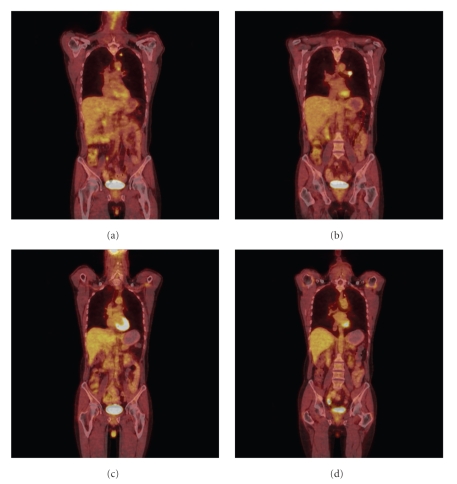
Coronal views of FDG-PET/CT scan obtained before (a)-(b) and after (c)-(d) treatment for primary NSCLC in patient with synchronous SBM. At diagnosis, the patient demonstrated hypermetabolic activity in a primary LUL tumor with mediastinal (a) and hilar (b)lymphadenopathy. Two years after treatment, including surgical resection, chemotherapy, and mediastinal RT, there is no evidence of hypermetabolic activity at the prior sites of thoracic involvement (c)-(d).

Staging evaluation was completed with whole-body, 18-fluoro-deoxyglucose (FDG)-positron emission tomography (PET)/computed tomography (CT) imaging, which demonstrated a hypermetabolic left upper lobe tumor and two hypermetabolic foci in the left para-aortic and suprahilar regions (Figures 2(a) and 2(b)). The FDG-PET/CT findings suggested a T1 primary tumor with N2 nodal disease. Given the absence of distant dissemination elsewhere, the control of intracranial disease (Figure 1(c)), and the patient's good KPS, it was decided that aggressive, multimodality therapy would be offered for management of the intrathoracic component of NSCLC. Video-assisted thorascopic surgery was performed for wedge resection of the LUL primary tumor and mediastinal lymph node sampling. Pathology showed poorly differentiated NSCLC, measuring 1.6 cm in greatest dimensions with negative resection margins. Two of 5 mediastinal lymph nodes in level V were positive, and 0 of 1 nodes in level IX. He subsequently received 5 cycles of platinum-based adjuvant chemotherapy. Repeat FDG-PET/CT after thoracic surgery and chemotherapy showed only mild hypermetabolic activity (maximum SUV of 1.65) in the LUL region, but no evidence of mediastinal uptake. Given the pathologic confirmation of N2 nodal disease in the surgical specimen, he was offered mediastinal RT in an effort to reduce the risk of regional nodal recurrence. He received 1.8 Gy daily fractions of RT to a total dose of 50.4 Gy to a target volume that encompassed the entire mediastinum (both ipsilateral and contralateral mediastinal nodal stations) and ipsilateral hilum.

Two years after completion of therapy, whole-body FDG-PET/CT imaging (Figures 2(c) and 2(d) and brain MRI (Figure 1(d)) have demonstrated no evidence of disease. The patient is without significant late toxicity from his diagnosis or treatment and has returned to work full time.

## 3. Discussion

This case serves as an example of the aggressive management of oligometastasis, a proposed, clinically significant disease state that exists between locoregional confinement and widespread metastatic disease [11]. The oligometastatic state may represent a window in which aggressive local intervention to sites of gross disease can result in long-term disease control. Multiple studies have demonstrated long-term survival for some patients after complete resection of brain, lung, or liver metastases [2, 3, 12–17]. While many of these patients had what are typically thought of as more “indolent” cancers (colorectal or breast), a subset of stage IV NSCLC patients will present with oligometastatic disease. Oh et al., in their study of 1284 patients with stage IV oligometastatic NSCLC, found that survival is influenced by the number of metastatic organ sites or the volume of oligometastatic disease in the brain or lung [18]. Therefore, the role of aggressive multimodality therapy in oligometastatic stage IV NSCLC warrants careful study and thoughtful consideration in clinical practice.

The use of aggressive, local therapy for SBM is supported by prospective trials that demonstrate a benefit for craniotomy or SRS for brain metastasis [3, 4]. While the addition of WBRT after surgery or SRS may not influence overall survival to a significant degree, it can improve local control, as supported by evidence from a prospective, randomized trial [19]. However, there are less data regarding management for the primary tumor in NSCLC patients who present with SBM. Retrospective studies have demonstrated benefit for aggressive local therapy for the primary lung tumor in this scenario [5–7, 20, 21]. Nodal status has been reported to be an important prognostic factor for NSCLC patients with SBM who receive aggressive treatment [5–8, 22], and treatment guidelines have suggested that NSCLC patients with involved mediastinal nodes are not to be considered for resection of the primary tumor [1]. However, the present case represents an instance of durable tumor control for a patient with synchronous SBM and N2 nodal disease. The long-term survival of NSCLC patients with mediastinal nodal disease and SBM has been reported infrequently in the literature [2], but our patient seems to have benefited from an aggressive approach.

The patient in the current report presented with an excellent performance status and was agreeable to an aggressive, multimodality treatment approach. Abrahams et al., in their report of 70 patients with NSCLC, found that the patients who benefited most from an aggressive treatment approach were those with a KPS of 100% [23]. Performance status was the most important prognostic factor in the Radiation Therapy Oncology Group recursive partitioning analysis of brain metastasis patients [24]. Given these considerations, performance status should be a primary consideration when considering aggressive treatment for patients with NSCLC and SBM.
